# Supplementary material for: Prediction of Residue-specific Contributions to Binding and Thermal Stability Using Yeast Surface Display
Source: Front Mol Biosci. 2022 Jan 21;8:800819. doi: 10.3389/fmolb.2021.800819 (PMC8814602; doi:10.3389/fmolb.2021.800819)
Supplement: Supplementary file 1 [file DataSheet1.PDF]

Prediction of residue-specific contributions to binding and thermal stability using yeast surface display

Shahbaz Ahmed[1], Munmun Bhasin[1], Kavyashree Manjunath[2], Raghavan Varadarajan[1*]

[1]Molecular Biophysics Unit, Indian Institute of Science, Bangalore-560012, India

[2]Institute for Stem Cell Science and Regenerative Medicine, Bangalore 560065, India

*Author for correspondence

Telephone: +91-80-22932612

Fax: +91-80-23600535

Email: varadar@iisc.ac.in

This file contains

Supplementary Figure S1-S14

Supplementary Table S1-S4

Supporting Information

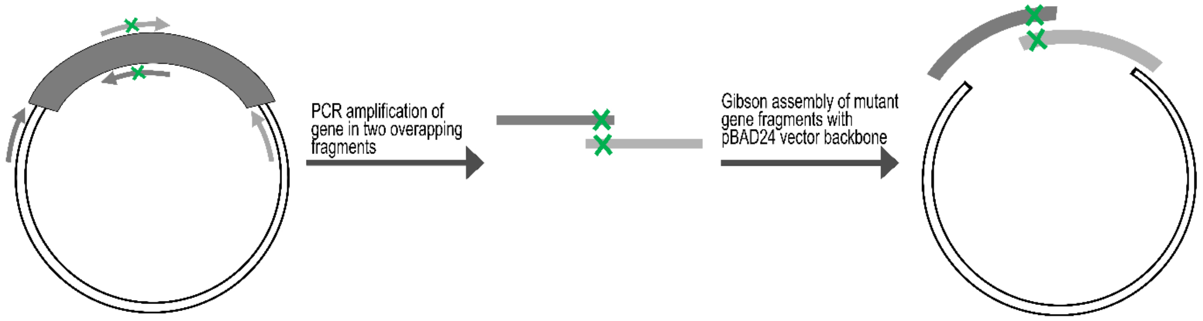

Supplementary Figure S1: Schematic representation of mutagenesis methodology used to introduce individual mutations in the *E.coli* expressed *ccdB* gene. The *ccdB* gene was amplified in two fragments using two sets of primers shown in light and dark grey. The location of the mutation is indicated by a green 'X'. The PCR amplified products were gel extracted and 3 fragment Gibson assembly was performed using NdeI and HindIII digested pBAD24 plasmid. The assembled products were transformed in *E.coli* Top10 GyrA cells and individual mutations were confirmed by Sanger sequencing.

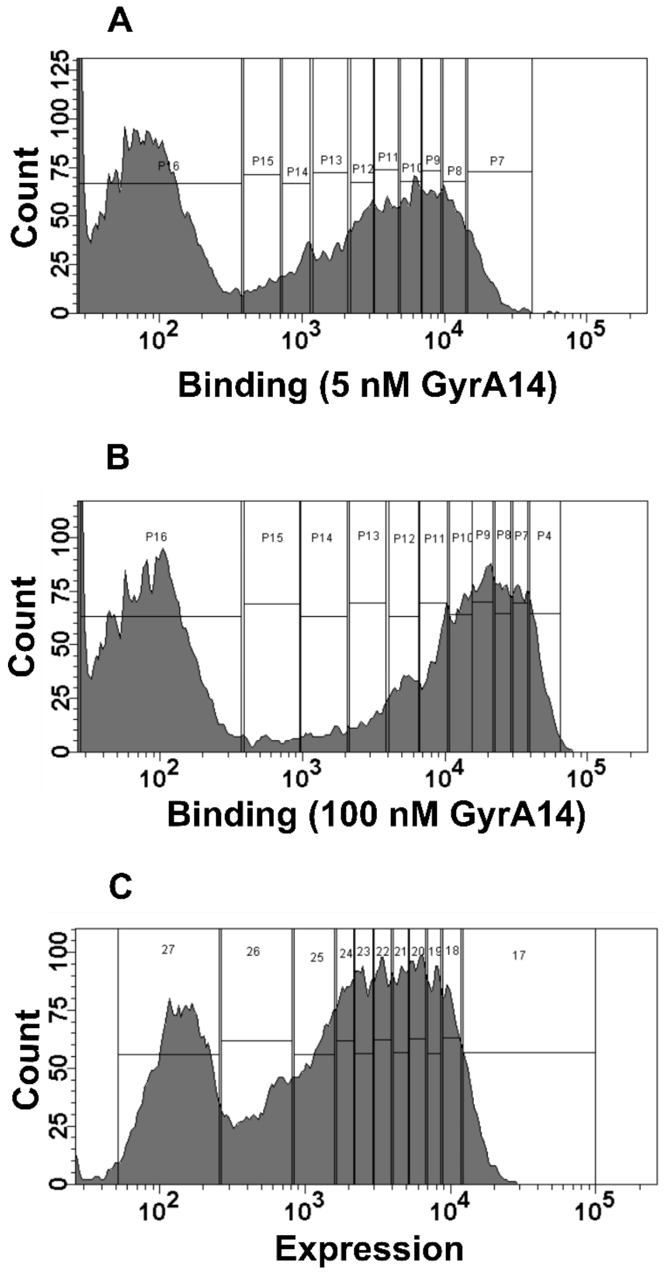

Supplementary Figure S2: FACS histograms for CcdB mutant library displayed on the yeast cell surface. Multiple vertical bins were used to sort the library according to (A) binding to 5 nM GyrA14, (B) binding to 100 nM GyrA14 and (C) expression of CcdB mutants. Each bin contained approximately equal numbers of cells.

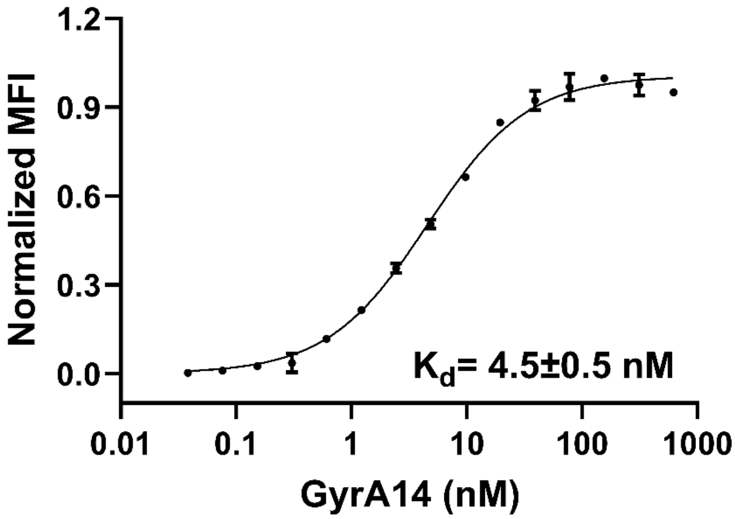

Supplementary Figure S3: Apparent K_d of interaction between CcdB and GyrA14 on yeast cell surface. WT CcdB protein was expressed on the yeast cell surface and MFI bind as a function of GyrA14 concentration was used to estimate the K_d. Error bars are from two biological replicates.

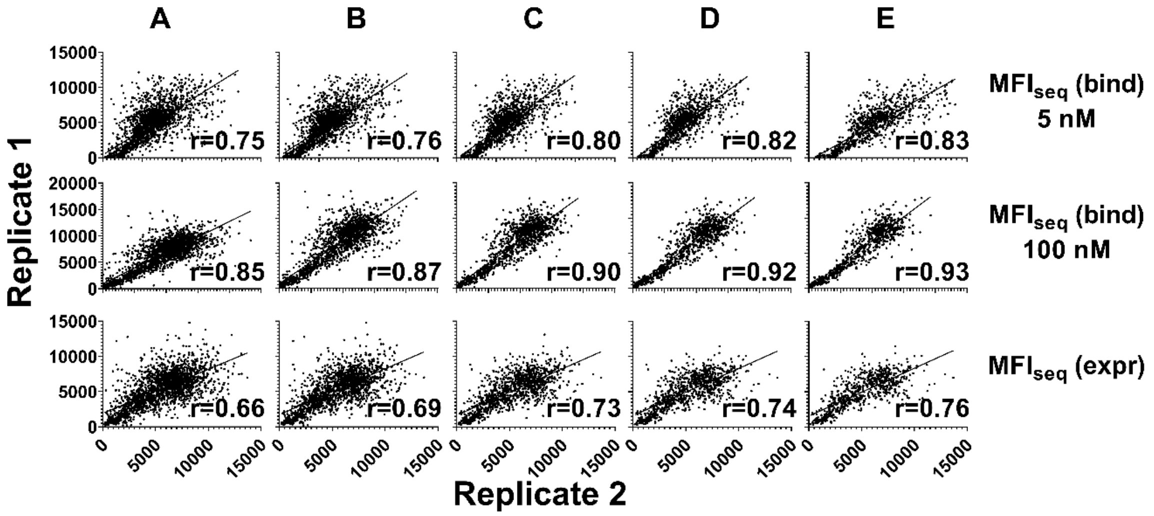

Supplementary Figure S4: Correlation of MFI_{seq} values between biological replicates obtained at different stringencies (sum of total reads of a mutant across all the bins of binding or expression histogram). The MFI_{seq} was calculated and a correlation between replicates was measured at stringencies of (A) 25 reads, (B) 50 reads, (C) 100 reads, (D) 150 reads, (E) 200 reads. Only mutants whose total read count exceeded the indicated stringency value in both replicates were considered for analysis. A read count of 50 was chosen for subsequent analyses, as increasing stringency further did not significantly improve the correlation and resulted in fewer mutants being selected. P <0.0001 for the significance of non-zero slope values in all the correlations.

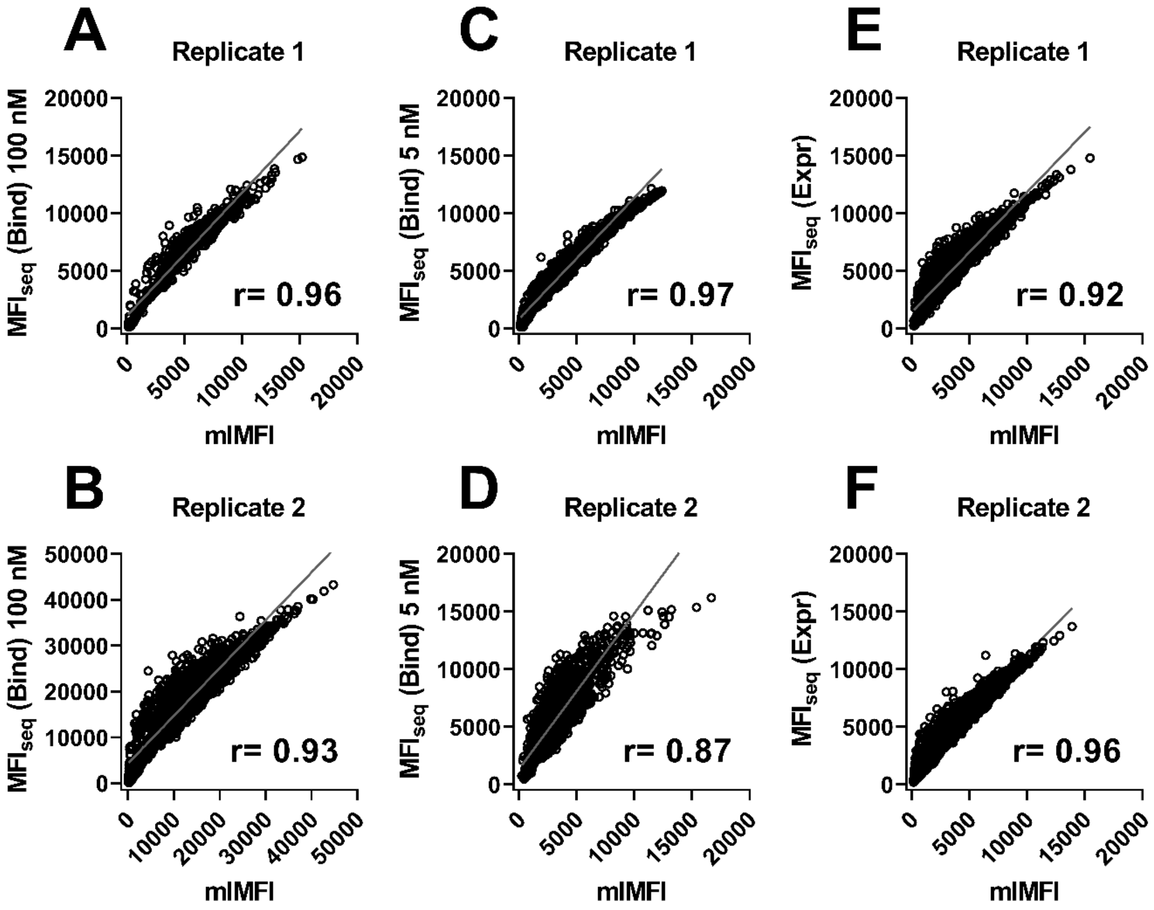

Supplementary Figure S5: Correlation between MFI of mutants calculated using Maximum Likelihood Method mlMFI using fitdistrplus R package and MFI_{seq}. A good correlation was found between mlMFI and MFI_{seq} for biological replicates at GyrA14 concentrations of 100 nM (A, B), 5 nM (C, D) and for cell surface expression (E, F). Using MFI_{seq} methodology we could calculate the MFI of binding for highly destabilizing as well as nonsense mutants, whereas with mlMFI, for several of these mutants it was not possible to calculate the MFI as all the reads were found in a single bin with the lowest MFI. $P < 0.0001$ for the significance of non-zero slope values in all the correlations.

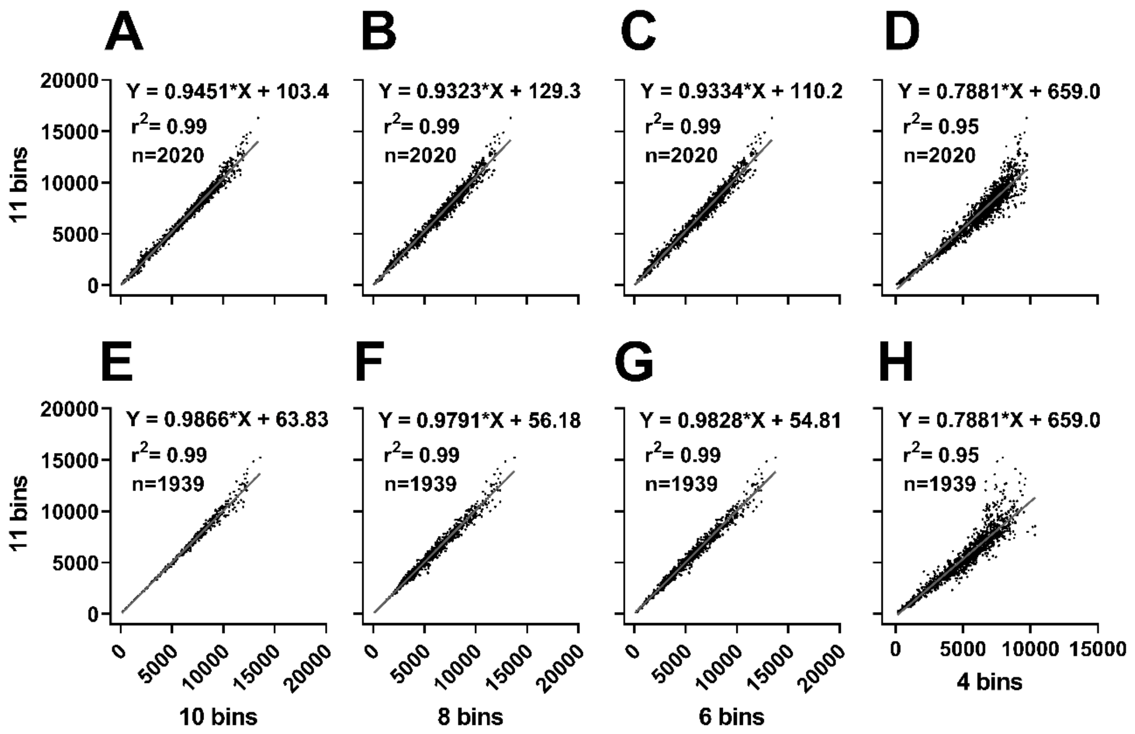

Supplementary Figure S6: Effect of number of bins on calculated MFI values. The MFI_{seq} (A-D) and mlMFI (E-H) of mutants was calculated using varying numbers of bins. The binding MFI of CcdB mutants was calculated using 11 bins (y-axis, all panels) and correlated with MFI calculated using 10 bins (A, E), 8 bins (B, F), 6 bins (C, G), 4 bins (D, H). Calculated values for 10, 8 and 6 bins are similar to those with 11 bins for both MFI_{seq} and mlMFI values. P <0.0001 for the significance of non-zero slope values in all the correlations.

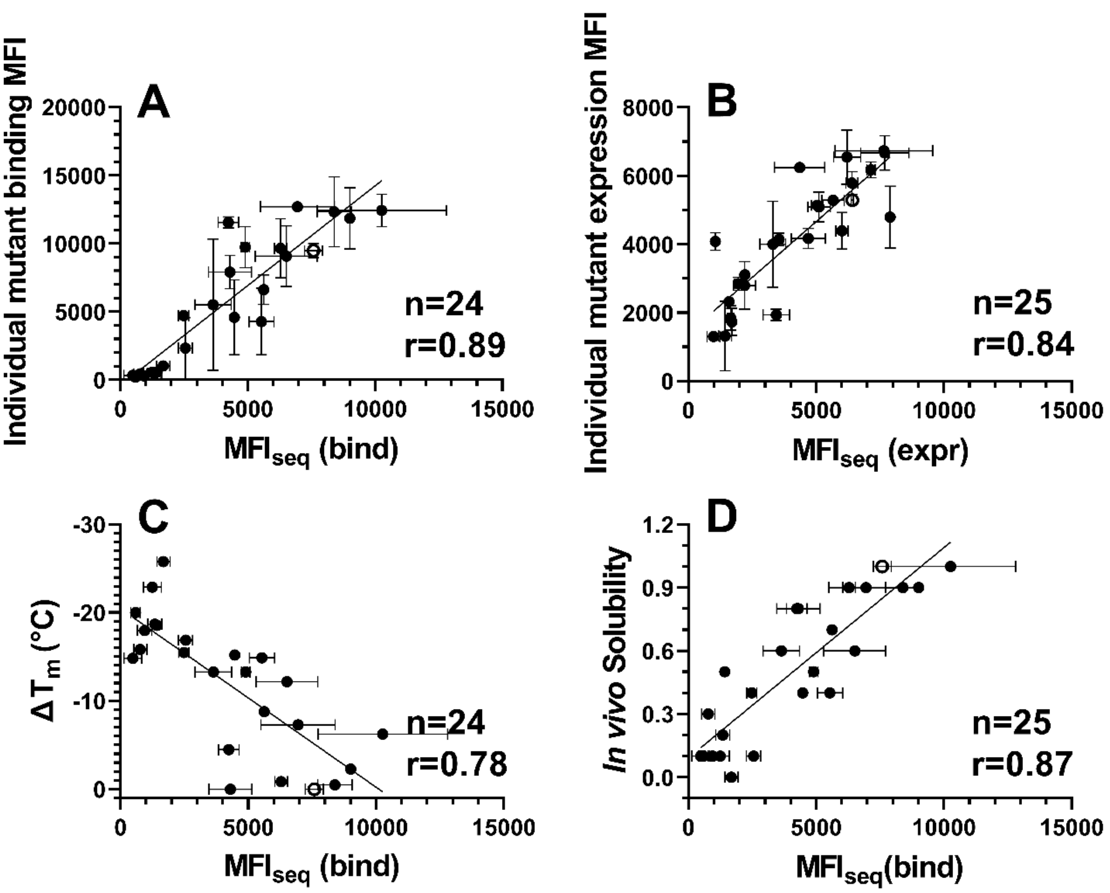

Supplementary Figure S7: MFI_{seq} correlates well with the MFI of individual mutants. Correlation between experimentally measured binding MFI from two biological replicates of individually analysed mutants and $MFI_{seq.}$ (A) MFI_{seq} (bind), (B) MFI_{seq} (expr). Correlation of MFI_{seq} (bind) with (C) *in vitro* thermal stability, (D) fractional *in vivo* solubility when expressed in *E.coli.* WT data is shown in open circles. P <0.0001 for the significance of non-zero slope values in all the correlations.

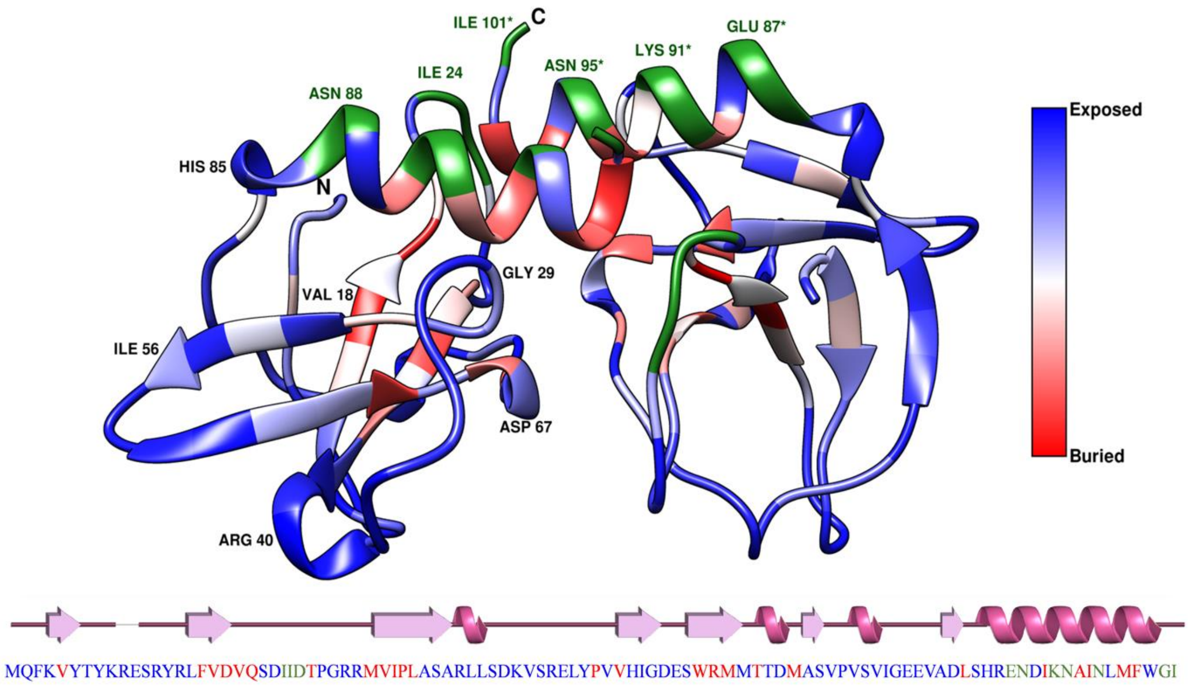

Supplementary Figure S8: Structure of homodimeric CcdB. Blue to red gradation represents increasing residue depth. Active-site residues of CcdB involved in binding DNA Gyrase are highlighted in green colour (PDB ID 1X75), with a few residues labelled for representation (* represents the residues from the second chain of the homodimer). Representative residue positions where individual mutants were characterized by YSD (Figure 1) are labelled in black. The secondary structure of CcdB, along with its amino acid sequence, is shown below the structure. In the sequence, red, blue and green represent buried, exposed non active-site, and active-site residues respectively.

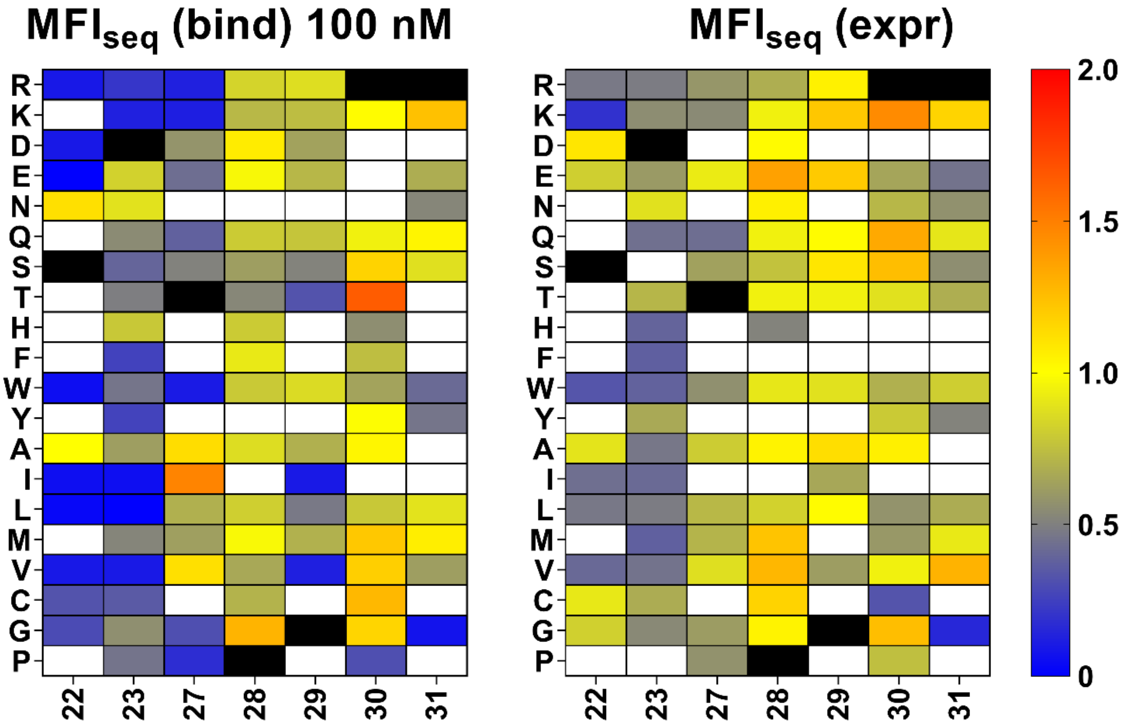

Supplementary Figure S9: Heat map of normalized binding and expression MFIs of GyrA14 non-interacting residues in the loop connecting β strands S2 and S3. Blue to red colour represents increasing normalized MFI_{seq} values, black represents the WT residue at the corresponding position. The loop residues 22, 23 and 27 have very high mutational sensitivity in the case of binding at 100 nM of GyrA14. However, expression is largely unaffected by mutation, indicating this region is important for CcdB interaction with GyrA14 even though there is no direct contact with GyrA14 at any of these residues.

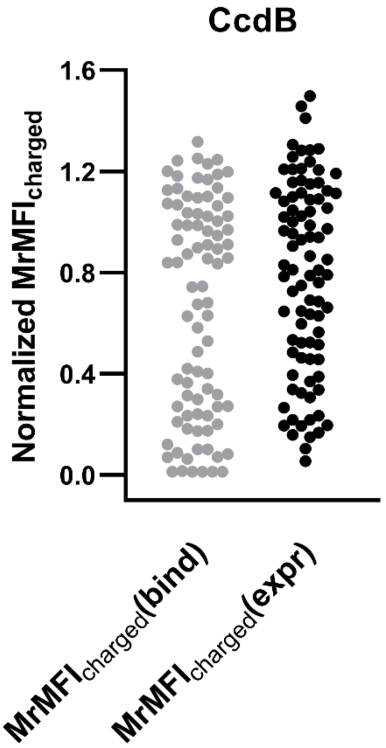

Supplementary Figure S10: Distribution of $MrMFI_{charged}$ for CcdB. Binding as well as expression $MrMFI_{charged}$ shows a bimodal distribution in CcdB. k-means clustering was used to calculate the mean and standard deviation of both distributions to differentiate buried and active-site residues.

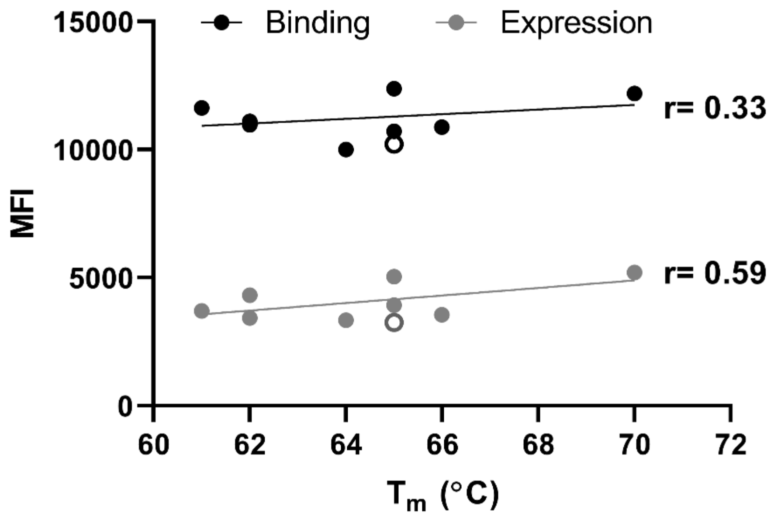

Supplementary Figure S11: Moderate correlation of MFI values with T_m for mutants with high values of both MFI_{seq} (bind) and MFI_{seq} (expr). WT is shown in open circles. P=0.13 and 0.42 for the significance of non-zero slope values in the correlations of expression and binding MFI with T_m.

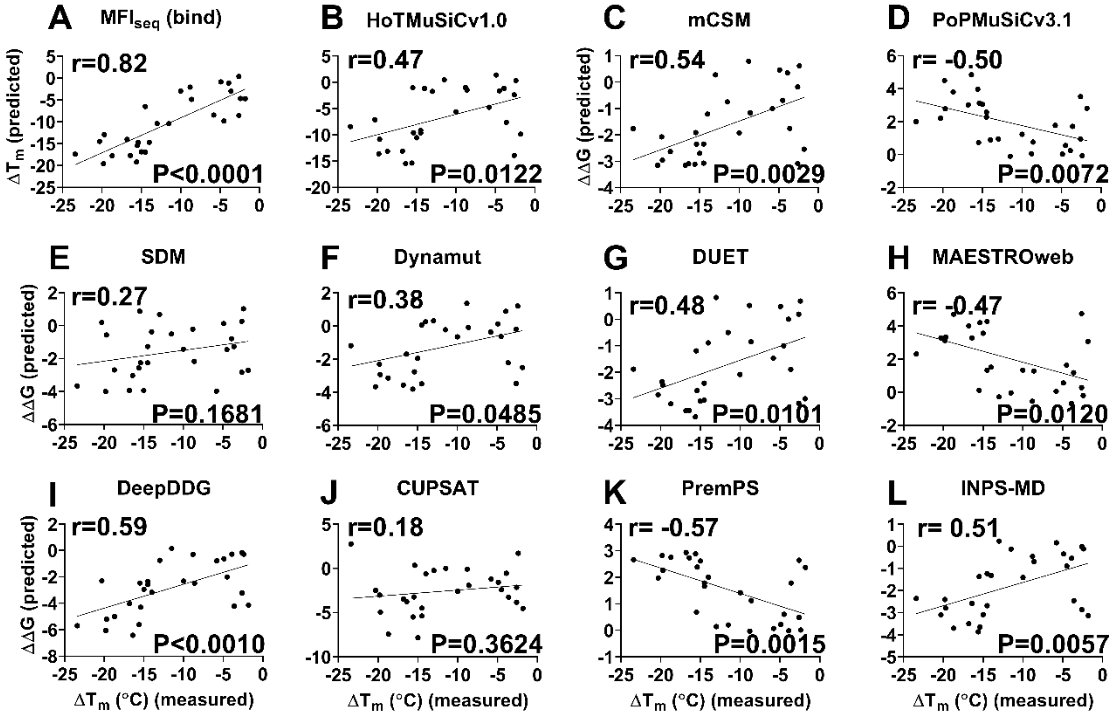

Supplementary Figure S12: Correlation between predicted and observed ΔT_m of CcdB mutants. The stability of CcdB mutants relative to WT was predicted by either (A) MFI_{seq} (bind) or (B-L) existing *in silico* tools. The best correlation was observed between MFI_{seq} (bind) predicted T_m and experimentally measured stability. Thermal stability predictions by *in silico* methods were best for DeepDDG, PremPS and mCSM. For plots with a positive slope, mutants with $\Delta\Delta G>0$ are predicted to be stabilizing, for plots with a negative slope, mutants with $\Delta\Delta G<0$ are predicted to be stabilizing. P values indicate the significance for non-zero slope values in all the correlations.

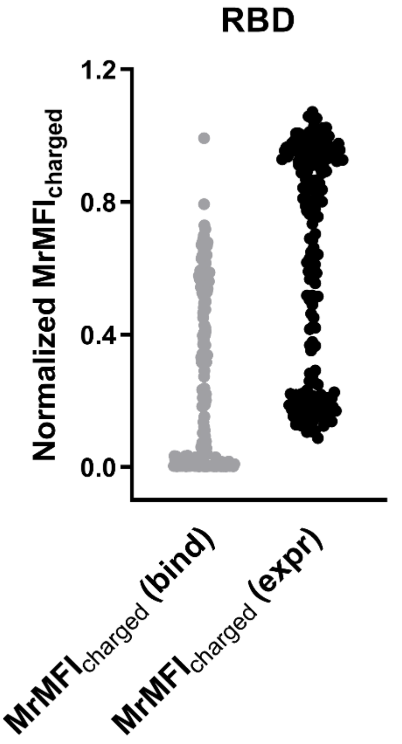

Supplementary Figure S13: Distribution of $MrMFI_{charged}$ of SARS-CoV-2 RBD. Normalized $MrMFI_{charged}$ for both expression and binding show a bimodal distribution similar to CcdB. k-means clustering was used to calculate the mean and standard deviation of both distributions to differentiate buried and active-site residues.

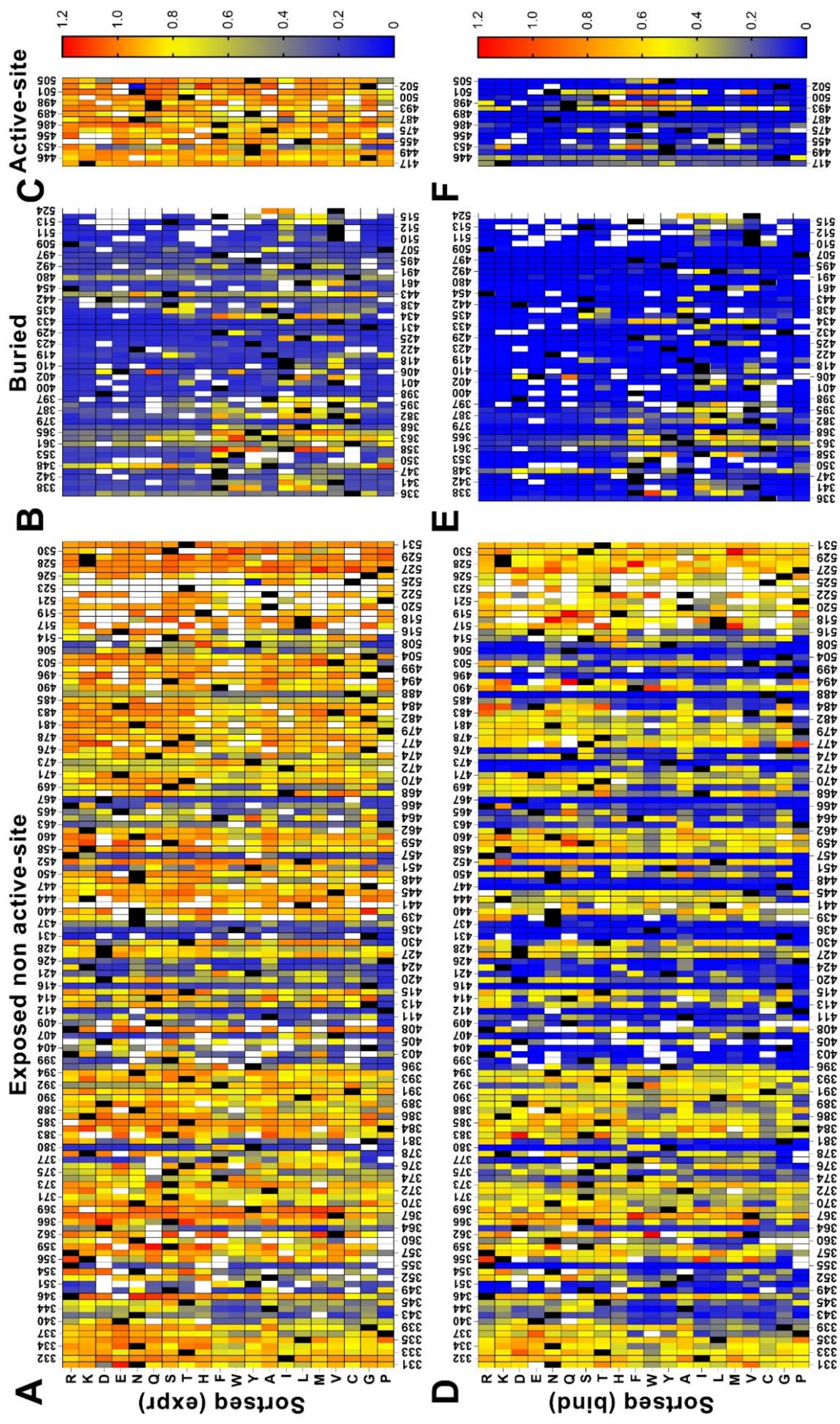

Supplementary Figure S14: Heatmap of normalized Sortseq MFI values for RBD mutants. Sortseq (expr) for (A) exposed non active-site residues, (B) buried residues and (C)

active-site residues. Sortseq (bind) at 100 nM ACE-2 for (D) exposed non active-site, (E) buried and (F) active-site residues. Exposed, buried (PDB ID:7KMH) and active-site (PDB ID:6M0J) residues are segregated based on the crystal structure. Blue to red colour represents increasing normalized Sortseq MFI values, black colour shows the WT residue at the corresponding position. White colour indicates that the mutant is not available.

Tables

Supplementary Table S1: Correlation coefficient (r) for MFI_{seq} values of individual mutants between two replicates at different stringencies. For stringencies of 50 reads or greater there is good correlation of both MFI_{seq} (bind) and MFI_{seq} (expr) between replicates.

Stringency	Experiment 1	Experiment 2	r
25 reads	MFI_{seq} (expr)	MFI_{seq} (expr)	0.66
	MFI_{seq} (bind) at 5 nM GyrA14	MFI_{seq} (bind) at 5 nM GyrA14	0.75
	MFI_{seq} (bind) at 100 nM GyrA14	MFI_{seq} (bind) at 100 nM GyrA14	0.85
50 reads	MFI_{seq} (expr)	MFI_{seq} (expr)	0.69
	MFI_{seq} (bind) at 5 nM GyrA14	MFI_{seq} (bind) at 5 nM GyrA14	0.76
	MFI_{seq} (bind) at 100 nM GyrA14	MFI_{seq} (bind) at 100 nM GyrA14	0.87
100 reads	MFI_{seq} (expr)	MFI_{seq} (expr)	0.73
	MFI_{seq} (bind) at 5 nM GyrA14	MFI_{seq} (bind) at 5 nM GyrA14	0.80
	MFI_{seq} (bind) at 100 nM GyrA14	MFI_{seq} (bind) at 100 nM GyrA14	0.90
150 reads	MFI_{seq} (expr)	MFI_{seq} (expr)	0.74
	MFI_{seq} (bind) at 5 nM GyrA14	MFI_{seq} (bind) at 5 nM GyrA14	0.82
	MFI_{seq} (bind) at 100 nM GyrA14	MFI_{seq} (bind) at 100 nM GyrA14	0.92
200 reads	MFI_{seq} (expr)	MFI_{seq} (expr)	0.76
	MFI_{seq} (bind) at 5 nM GyrA14	MFI_{seq} (bind) at 5 nM GyrA14	0.83
	MFI_{seq} (bind) at 100 nM GyrA14	MFI_{seq} (bind) at 100 nM GyrA14	0.93

Supplementary Table S2: Correlation coefficient (r) for MFI_{seq} values of individual mutants with CcdB structural parameters calculated from the WT structure (PDB ID: 3VUB). All correlations are improved when MFI_{seq} values are averaged (MrMFI) over all mutants at the given residue.

Parameter 1	Parameter 2	r
Depth	MFI_{seq} (bind)at 5 nM GyrA14	-0.29
	MrMFI (bind)at 5 nM GyrA14	-0.33
Depth	MFI_{seq} (bind) at 100 nM GyrA14	-0.32
	MrMFI (bind) at 100 nM GyrA14	-0.44
Depth	MFI_{seq} (expr)	-0.45
	MrMFI (expr)	-0.58
% Accessibility	MFI_{seq} (bind)at 5 nM GyrA14	0.27
	MrMFI (bind) at 5 nM GyrA14	0.30
% Accessibility	MFI_{seq} (bind) at 100 nM GyrA14	0.27
	MrMFI (bind) at 100 nM GyrA14	0.33
% Accessibility	MFI_{seq} (expr)	0.37
	MrMFI (expr)	0.44
RankScore	MFI_{seq} (bind)at 5 nM GyrA14	-0.37
	MrMFI (bind) at 5 nM GyrA14	-0.37
RankScore	MFI_{seq} (bind)at 100 nM GyrA14	-0.47
	MrMFI (bind) at 100 nM GyrA14	-0.61
RankScore	MFI_{seq} (expr)	-0.53
	MrMFI (expr)	-0.57

Supplementary Table S3

The specificity, sensitivity and accuracy of exposed non active-site, buried site and exposed active-site residue identification solely from MFI_{seq} or Sortseq.

	**Specificity[a]**		**Sensitivity[a]**		**Accuracy[a]**	
**Proteins**	CcdB	RBD	CcdB	RBD	CcdB	RBD
**Exposed non-active site**	0.98	0.84	0.82	0.74	0.92	0.80
**Buried**	1	0.87	0.76[b]	0.70[b]	0.93	0.81
**Exposed active-site**	0.97	0.98	0.88	0.78	0.95	0.96

[a]The specificity, sensitivity and accuracy were calculated using the following formulae, where TN, TP, FN and FP correspond to true negative, true positive, false negative and false positive respectively.

Specificity = $\frac{TN}{TN+FP}$

Sensitivity = $\frac{TP}{TP+FN}$

Accuracy = $\frac{TP+TN}{TP+TN+FP+FN}$

[b]Sensitivity increases to 0.79 and 0.87 for CcdB and RBD respectively if false negative Gly residues are not considered.

Supplementary Table S4

The specificity, sensitivity and accuracy for buried site identification solely from protein sequence using the PROF predictor (Rost and Sander, 1994).

**Protein**	**Specificity**	**Sensitivity**	**Accuracy**
CcdB	0.73	0.87	0.77
RBD	0.81	0.71	0.78
Aminoglycoside kinase	0.82	0.71	0.79
BRCA1 RING domain	0.83	0.55	0.77
Gal4 (DBD)	0.96	0.89	0.95
GB1 (IgG-binding domain)	0.93	0.60	0.87
Hsp90 (ATPase domain)	0.92	0.70	0.87
NUDT15	0.83	0.67	0.79
Pab1 (RRM domain)	0.88	0.61	0.81
PSD (pdz3 domain)	0.93	0.64	0.84
TEM1 β- lactamase	0.85	0.74	0.81
Ubiquitin	0.92	0.88	0.91
**AVERAGE**	**0.87**	**0.71**	**0.83**

[a]The specificity, sensitivity and accuracy were calculated using the following formulae, where TN, TP, FN and FP correspond to true negative, true positive, false negative and false positive respectively.

Specificity = $\frac{TN}{TN+FP}$

Sensitivity = $\frac{TP}{TP+FN}$

Accuracy = $\frac{TP+TN}{TP+TN+FP+FN}$

References

Rost, B., and Sander, C. (1994). Combining evolutionary information and neural networks to predict protein secondary structure. *Proteins Struct. Funct. Bioinforma.* 19, 55–72. doi:10.1002/prot.340190108.
